# Supplementary material for: A Meta-Analysis on Quantitative Sodium, Potassium and Chloride Metabolism in Horses and Ponies
Source: Animals (Basel). 2025 Jan 13;15(2):191. doi: 10.3390/ani15020191 (PMC11758655; doi:10.3390/ani15020191)
Supplement: Supplementary file 1 [file animals-15-00191-s001.zip › animals-3333451-supplementary.pdf]

Figure S1. Flow diagram for study search and selection based on Preferred Reporting Items for Systematic Reviews and Meta-Analyses (PRISMA).

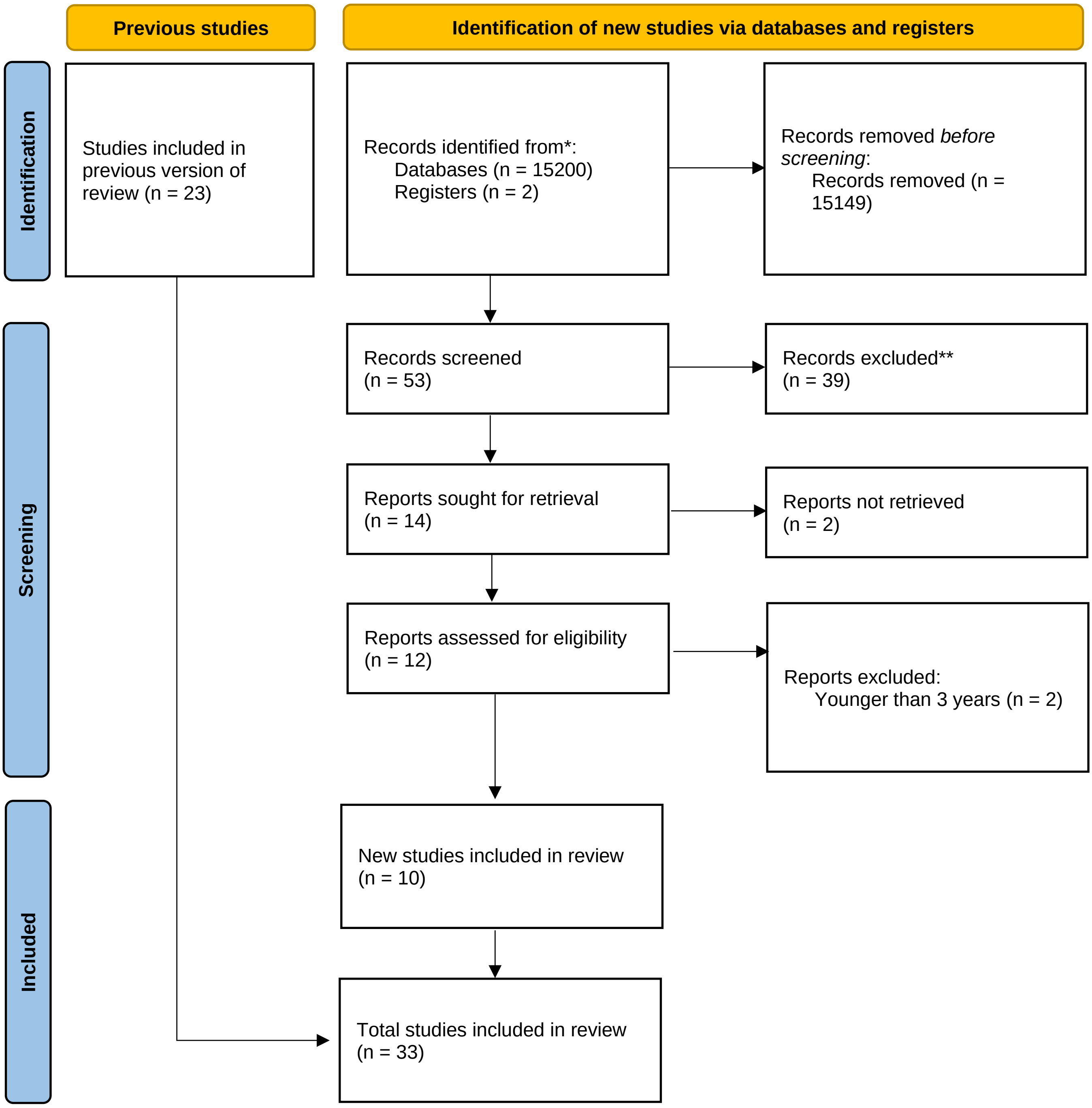

Table S1. Number of included studies and participants for each graph.

Mineral	Subject	Number of studies	Number of participants
Na	Figure 1	31	369
	Figure 2	31	369
	Figure 3	16	110
	Figure 4	24	225
	Figure 5	24	225
	Figure 6	5	20
K	Figure 7	27	298
	Figure 8	27	298
	Figure 9	14	97
	Figure 10	22	171
	Figure 11	21	162
	Figure 12	5	20
Cl	Figure 13	11	65
	Figure 14	11	59
	Figure 15	5	33
	Figure 16	12	71
	Figure 17	11	62
	Figure 18	4	10
